# Adult Human Glia, Pericytes and Meningeal Fibroblasts Respond Similarly to IFNy but Not to TGFβ_1_ or M-CSF

**DOI:** 10.1371/journal.pone.0080463

**Published:** 2013-12-05

**Authors:** Amy M. Smith, E. Scott Graham, Sheryl Xia Feng, Robyn L. Oldfield, Peter M. Bergin, Edward W. Mee, Richard L. M. Faull, Maurice A. Curtis, Mike Dragunow

**Affiliations:** 1 Department of Pharmacology and Clinical Pharmacology, The University of Auckland, Auckland, New Zealand; 2 Gravida - National Research Centre for Growth and Development, Auckland, New Zealand; 3 Department of Anatomy, The University of Auckland, Auckland, New Zealand; 4 Centre for Brain Research, The University of Auckland, Auckland, New Zealand; 5 Lab Plus, Auckland, New Zealand; 6 Auckland City Hospital, Auckland, New Zealand; Virginia Commonwealth University, United States of America

## Abstract

The chemokine Interferon gamma-induced protein 10 (IP-10) and human leukocyte antigen (HLA) are widely used indicators of glial activation and neuroinflammation and are up-regulated in many brain disorders. These inflammatory mediators have been widely studied in rodent models of brain disorders, but less work has been undertaken using human brain cells. In this study we investigate the regulation of HLA and IP-10, as well as other cytokines and chemokines, in microglia, astrocytes, pericytes, and meningeal fibroblasts derived from biopsy and autopsy adult human brain, using immunocytochemistry and a Cytometric Bead Array. Interferonγ (IFNγ) increased microglial HLA expression, but contrary to data in rodents, the anti-inflammatory cytokine transforming growth factor β1 (TGFβ_1_) did not inhibit this increase in HLA, nor did TGFβ_1_ affect basal microglial HLA expression or IFNγ-induced astrocytic HLA expression. In contrast, IFNγ-induced and basal microglial HLA expression, but not IFNγ-induced astrocytic HLA expression, were strongly inhibited by macrophage colony stimulating factor (M-CSF). IFNγ also strongly induced HLA expression in pericytes and meningeal fibroblasts, which do not basally express HLA, and this induction was completely blocked by TGFβ_1_, but not affected by M-CSF. In contrast, TGFβ_1_ did not block the IFNγ-induced increase in IP-10 in pericytes and meningeal fibroblasts. These results show that IFNγ, TGFβ_1_ and M-CSF have species- and cell type-specific effects on human brain cells that may have implications for their roles in adult human brain inflammation.

## Introduction

Although the brain was long thought to have limited immunological activity, it is now appreciated that substantial immune activity occurs in the brain at a homeostatic level as well as during disease [1]. Markers of immune activation are ubiquitously used to track disease progress, correlate with symptomology, and have become a major target for disease therapies [2]. Brain-resident microglia are immune cells of myeloid origin. Microglia are the predominant antigen-presenting cell types of the brain and they perform a variety of functions including phagocytosis of debris, production of signalling molecules and monitoring extracellular ion levels [3]. Immune surveillance of the CNS is important for many homeostatic processes. However, neuroinflammation is thought to contribute to the pathogenesis of many neurological disorders [4]–[6]. A complete understanding of the phenotype of microglia in the adult human brain is still lacking as there is evidence that human adult microglia are different to fetal microglia and blood monocytes [7], [8]. Dystrophic microglia have been identified in the aged human brain and ‘microglial senescence’ is a possible contributor to neurological decline [9], [10]. Furthermore, immune responsiveness changes with age and over time microglia may become increasingly activated [11]. The “activated” microglial phenotype can be assessed in multiple ways, including expression of proteins involved in functional activities such as antigen presentation, morphological changes, and functional activation such as production of cytokines and chemokines.

Other cells apart from microglia have immune roles in the brain. Astrocytes perform many homeostatic functions which impact on immune activity in the CNS, for example maintaining BBB integrity, glutamate recycling, and potassium buffering [1]. Astrocytes also have many direct roles in the innate immunity of the CNS. They express innate immune receptors (e.g. TLR3 and CXCR3) and secrete soluble mediators which affect immune responses (e.g. TGFβ_1_, IL-6, and IL-10) [12], [13]. Astrocyte immune activity has been shown to play a specific role in several diseases including Alzheimer's disease (AD) [14] and epilepsy [15], partially through upregulated expression of pro-inflammatory cytokines.

Many other cells contribute to immune responses in the CNS, including cells at the blood-brain barrier such as pericytes [16]–[18], perivascular macrophages, perivascular mesenchymal stem cells [19] and other cells adjacent to the CNS parenchyma such as meningeal fibroblasts of the leptomeninges [1], [20]. We have previously identified and characterized a population of fibroblast-like cells in cultures of adult human brain tissue that express the fibroblast markers prolyl-4-hydroxylase and fibronectin [21], [22]. These cells do not express markers of microglia or astrocytes, and are likely to be of neurovascular origin as they also express markers of pericytes [19], [22]. Overall, this cell population expresses the fibroblast and pericyte markers prolyl-4-hydroxylase, vimentin, nestin, α-smooth muscle actin and platelet-derived growth factor receptor-β [22]. We refer to these cells as “pericytes”, in-line with the current literature [19], [22]. We show here that this cell population exhibits distinct immune characteristics. These cells are likely distributed throughout the CNS in ideal locations for immune interaction, both with cells of the periphery and of the CNS [19].

An essential aspect of neuroinflammation is cross-talk between different cells of the immune and central nervous systems via cell surface proteins and secreted molecules. Human leukocyte antigen (HLA) is a cell surface antigen presentation protein. HLA-DP, DQ and DR classes present extracellular antigens to T cells and are the human-specific versions of the class II Major Histo-Compatibility (MHC) complex in vertebrates. There are numerous reports of increased HLA and MHC class II expression with brain injury and disease processes in both rodent models and human post-mortem brain tissue [23]. For example, an increased number of HLA-DR positive microglia have been found in epileptic hippocampus compared to control human brain [24] and progressive accumulation and correlation to disease has been found for HLA-positive microglia in Huntington's and Alzheimer's disease brain tissue [25], [26]. MHC class II expression is increased in response to neuronal injury [27] and dense focal clusters of HLA-DR immunoreactivity are visible at senile plaques in AD gray matter [28]. While microglia are the predominant resident cell type in the brain to express HLA both *in vitro* and *in situ*, Styren et al. (1990) have shown that astrocytes in control and AD brains can also express HLA-DR [28]. Given its upregulation in so many diseases, the regulation of HLA in the adult human brain is of great interest. Substantial research has been conducted on the regulation of HLA expression [29], and it has become apparent that there can be species and cell type specific differences in its regulation.

A major cytokine known to influence HLA expression is the T-cell cytokine Interferon-y (IFNy). IFNy acts through the MHC Class II Transactivator (CIITA), the master regulator of MHC II gene expression [30]. Two other molecules which can affect HLA expression are Transforming Growth Factor β_1_ (TGFβ_1_) and Macrophage Colony-Stimulating Factor (M-CSF). The predominantly anti-inflammatory cytokine TGFβ_1_ has been shown to counteract the upregulation of HLA by IFNy via inhibition of the expression of IFNy-induced CIITA mRNA [30]–[33]. The effect of M-CSF on basal and IFNy-induced HLA-DR has previously been investigated in human fetal astrocytes and microglia [34], where it was found that M-CSF reduced HLA-DR in microglia but not astrocytes [34]. However, the relevance of these findings to the adult human brain is still to be determined.

The cytokine IFNy not only affects immune responses by inducing expression of cell surface proteins but also produces changes in glial cytokine and chemokine production. Interferon gamma-induced protein 10 (IP-10; CXCL10) is produced by a variety of cells in the brain in response to IFNy. This chemokine functions in selective trafficking of leukocytes, migration of glia and proliferation of various cell types [35]. IP-10 binds the G protein–coupled receptor CXCR3 [36]. CXCR3 expression has been reported in the developing human brain [37] and in human and rodent cultured microglia and astrocytes [38], [39].

IP-10 and CXCR3 have been shown to be increased in several neurological disease states [13]. IP-10 plays a particular role in viral infection as it is induced by the T-cell anti-viral cytokine IFNy. As such, IP-10 was found to be elevated in the CSF from patients with viral meningitis [40]. In a study of human brain tissue, IP-10 immunoreactivity was not detected in HIV-negative brains, but was present in HIV-positive brains and further found to be induced in human neurons by HIV infection *in vitro* [41]. Multiple sclerosis (MS) is an autoimmune demyelinating disease which involves a large recruitment of lymphocytes into the brain parenchyma. CXCR3-positive T cells are increased in blood [42] and brain tissue [43] of MS patients compared with healthy controls. Blocking IP-10 in the experimental autoimmune encephalomyelitis (EAE) mouse model of MS reduces the severity of the disease and the number of pathogenic T-cells in the inflamed CNS [40]. IP-10 is a common feature of other neurological conditions including AD [44] and glioma [45]. It is clear that IP-10 plays a profound role in neurological disease and the extracellular factors regulating IP-10 expression in the adult human brain require further investigation.

Our study investigates the effects of cytokines IFNγ, TGFβ_1_ and M-CSF on adult human glial inflammatory mechanisms, namely the inducible expression of HLA-DP, DQ, DR and production of cytokines and chemokines by microglia, astrocytes, pericytes and meningeal fibroblasts. Parts of this work were presented to the XI International Congress of Neuroimmunology (ISNI) in Boston 2012 with an abstract published in the Journal of Neuroimmunology volume 253 page 115 (2012).

## Methods

### Tissue

Biopsy human temporal lobe tissue was from subjects receiving surgery for intractable epilepsy, and the research was approved by the Northern Regional Ethics Committee. All biopsy specimens were from temporal lobe epilepsy cases (n = 10) with varying degrees of mesial temporal sclerosis (neuropathological grade 3–4, where grade 4 is maximal severity). Autopsy adult human brain tissue (temporal lobe) from a range of neurologically diseased (Alzheimer's, n = 2; Huntington’s, n = 1; and Parkinson’s disease, n = 1) and normal individuals (n = 3) was obtained through the Neurological Foundation of New Zealand Human Brain Bank (University of Auckland Human Participants Ethics Committee). Informed written consent was obtained in all cases.

### Human glial cell isolation and culture

Cells were obtained from adult human brain (middle temporal gyrus) tissue as previously described [21], [46], [47], and were cultured for ∼1 week prior to plating for experiments at 50,000 cells/ml in 96-well plates. This initial passaging of cells consisted of a mixed glial culture containing microglia, astrocytes and pericytes, as has been previously characterised [48]. All cultures were validated for cell phenotypes as per Gibbons *et al.* [21]. The percentage of different cell types varies between cultures, with an average of 13.2+/−1.3% PU.1-positive microglia, and 1.1+/−0.02% GFAP-positive astrocytes (mean +/− SEM, n = 3 cases) [48]. To obtain cultures of pericytes only, 3 or 4 subsequent passages were made (roughly 1 week apart, when cells had reached ∼90% confluence) and the negligibly dividing microglia and astrocytes were no longer present, as determined by immuno-labelling for microglia (PU.1) and astrocytes (GFAP) [21], [22].

### Leptomeningeal explant cultures

To study the inflammatory role of meningeal fibroblasts, leptomeninges (from the same tissue as above) covering the middle temporal gyrus was carefully removed from underlying tissue using forceps. Small pieces of leptomeningeal tissue, ∼2×3 mm, were placed into wells of a 6-well plate with ∼850 µl (not so much that they were floating, but enough to surround them with nutrients) DMEM/F12 media supplemented with 10% fetal bovine serum (FBS), 1% Penicillin-Streptomycin-Glutamine (Gibco BRL) (final concentrations: penicillin (100 U/ml), streptomycin (100 µg/ml) and L-glutamine (0.29 mg/ml)). Half the volume of media was changed twice in the first week, and then a full media change was done every 3–4 days. Cells started to grow out of the tissue after ∼1 week. Leptomeningeal explants were passaged by moving to a new plate with forceps. For cytokine treatment, the explants were moved to a 24-well plate for 2 weeks to generate cells. The explants were then passaged into a new plate and the cells in the 24-well plate were left for 2 days before beginning cytokine treatment.

### Cytokine treatment

Mixed primary human glial cell cultures were treated in 96-well plates. 1 µl cytokine was added to 100 µl media. Cells were treated with 1 ng/ml IFNy (in PBS with 0.1% BSA) at 0 and 48 h. Total time of IFNy treatment was 96 h. Cells were pre-treated with 10 ng/ml TGFβ_1_ (in 1 mM citric acid pH 3 with 0.1% BSA) or 25 ng/ml M-CSF (in H_2_O) at 0, 24 and 48 h. The last pre-treatment (at 48 h) was given at the same time as the first IFNy treatment.

### Immunocytochemistry

Immunocytochemsitry was performed on cells as previously described using the following primary antibody and biotinylated secondary antibodies (see Table 1) [49].

**Table 1:** Antibodies used for immunocytochemistry.

Antibody	Company	Catalogue #	Dilution
Mouse anti-HLA-DP, DQ, DR	Dako	M0775	1∶500
Rabbit anti-PU.1	Cell Signaling	2258	1∶500
Mouse anti-CD45	Abcam	ab8216	1∶500
Mouse anti-GFAP	Dako	Z0334	1∶5000
Rabbit anti-IP-10	Abcam	ab9807	1∶500
Goat anti-rabbit IgG Alexa Fluor® 594	Invitrogen	A11012	1∶500
Goat anti-mouse IgG Alexa Fluor® 488	Invitrogen	A11001	1∶500
Goat anti-mouse IgG Alexa Fluor® 594	Invitrogen	A11005	1∶500
Goat anti-rabbit IgG Alexa Fluor® 488	Invitrogen	A11008	1∶500

### Quantitative image analysis of cell number, protein expression and microglial morphology

Immunocytochemical and morphological observations have been quantified using the Discovery-1 microscope (Molecular Devices) and Metamorph image analysis system as previously detailed and described [50], [51]. Microglial morphology was quantified as previously described [49].

### Quantitative cytokine and chemokine measurement

Conditioned media from experiments was collected after 96 h IFNy treatment. The media was filtered using a 0.2 µm filter (Pall Life Sciences) and stored at −80°C until use. A Cytometric Bead Array (B.D Biosciences) was performed according to the manufacturer's instructions using a FACSAria II flow cytometer (B.D Biosciences) [52].

### Statistical analysis

Data from representative experiments are displayed as mean ± standard error of the mean (SEM). Cells from at least 6 different individuals were used for experiments, except for quantitative cytokine/chemokine analysis for which 3 biopsy cases were used. The F-test and Bartlett's test were used to check for equal variances. Statistical analysis was carried out using t-tests and one-way ANOVA with Tukey's multiple comparison test where variances were equal, and the equivalent non-parametric test was used in cases of unequal variance (Mann Whitney test or Kruskal-Wallis test with Dunn's multiple comparison test). Statistically significant differences were set at P<0.05. Significant differences from vehicle (no cytokine treatment) are indicated.

## Results

Meningeal fibroblasts were prepared from leptomeningeal explants, and dissociated cultures comprising pericytes, microglia and astrocytes were prepared as previously described [21], [46], [48].

### Microglial expression of HLA-DP, DQ, DR is increased by IFNy and reduced by M-CSF but not by TGFβ_1_

Microglia are the predominant HLA-DP, DQ, DR-expressing cell type in human adult mixed glial cultures. Microglia from different cases express differing basal amounts of HLA-DP, DQ, DR. From 10 biopsy cases, 5 had high basal microglial HLA expression, 4 had moderate expression and 1 had low HLA expression (Table 2). Heterogeneity in HLA expression could not be explained by drug use or degree of sclerosis alone. We also did not observe any differences in HLA expression relating to differences in cellular composition of the cultures.

**Table 2:** Levels of HLA protein expression differ between biopsy cases.

Case Number	Microglia	Astrocytes	Pericytes
1	High	High	None
2	High	High	None
3	High	Moderate	None
4	High	Moderate	None
5	Moderate	None	None
6	Low	Low	None
7	Moderate	Low	None
8	Moderate	Low	None
9	Moderate	None	None
10	High	Moderate	None

In adult human mixed glial cultures, microglia and astrocytes express variable basal levels of HLA (qualitatively assessed by proportion of positive cells and intensity of staining). However, HLA expression is not observed on untreated brain-derived pericytes in culture.

**Figure 1:**
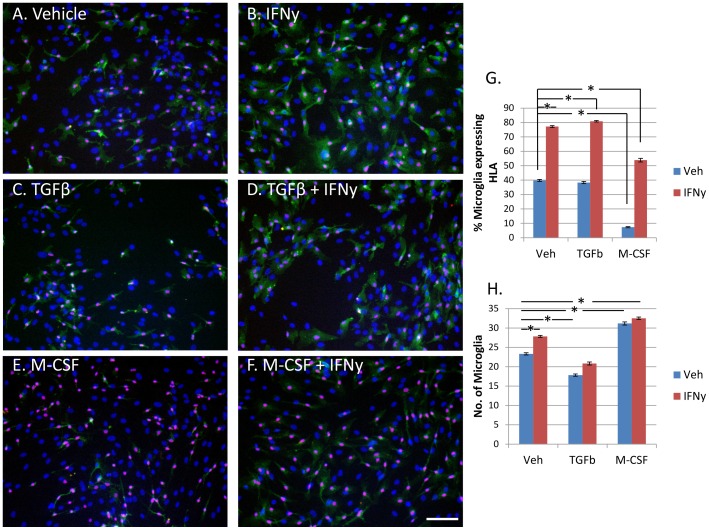
Microglial expression of HLA-DP, DQ, DR is increased by IFNy, not changed by TGFβ_1_, and reduced by M-CSF. A) Adult human PU.1+ve microglia (pink) express variable levels of HLA-DP, DQ, DR (green) in basal conditions without any treatment. All nuclei are labelled with Hoechst (blue). B) IFNy (1 ng/ml, 96 h) increased microglial expression of HLA-DP, DQ, DR, as well as HLA-DP, DQ, DR expression by astrocytes and pericytes in the mixed glial culture. C) TGFβ_1_ (10 ng/ml) did not affect microglial HLA-DP, DQ, DR expression alone, or when enhanced by IFNy treatment (D). E) M-CSF (25 ng/ml) reduced basal HLA-DP, DQ, DR expression in microglia and also decreased IFNy-enhanced HLA-DP, DQ, DR expression in microglia (F). Scale bar = 100 µm. G) A significant increase in percentage of HLA-positive microglia is found with IFNy treatment. No change in microglial HLA-DP, DQ, DR expression is seen for TGFβ_1_ treatment, but M-CSF significantly reduces microglial HLA-DP, DQ, DR protein expression (N = 12). H) The number of microglia per image, as measured by PU.1-immunopositive cells, is significantly increased by IFNy and M-CSF. However, TGFβ_1_ significantly reduces microglial cell number (N = 12).

HLA expression in adult human microglia was increased by exposure to IFNy (1 ng/ml), regardless of basal levels of expression (Fig. 1A and B). Adult human glial cultures were immunostained for the microglial transcription factor PU.1 and the percentage of HLA-immunopositive microglia was found to significantly increase with IFNy (Fig. 1G). The number of HLA-immunopositive microglia and the intensity of HLA expression were both increased by IFNy.

Contrary to the literature on rodent studies, TGFβ_1_ (10 ng/ml) treatment of human adult microglia did not reduce (or enhance) IFNy-induced HLA expression. Furthermore, no effect of TGFβ_1_ was observed for basal (vehicle-treated) microglial HLA expression (Fig. 1C, D and G).

We have previously reported that M-CSF-treated adult human microglia have reduced expression of HLA compared to vehicle-treated microglia [49]. Here we further report that M-CSF (25 ng/ml) in combination with IFNy significantly reduced the IFNy-mediated increase in microglial HLA expression (Fig. 1E, F and G).

### Microglial cell number is increased by IFNy and M-CSF, but reduced by TGFβ_1_

We have previously reported an increase in microglia number following M-CSF treatment [49]. Despite increased numbers of microglia we found simultaneously reduced HLA expression (Fig. 1G and H). IFNy was also found to slightly increase microglia number compared to vehicle. However, the increase in microglial cell number produced by IFNy was not as great as for M-CSF (Fig. 1H). Although TGFβ_1_ did not influence microglial expression of HLA, it did significantly reduce microglial cell number (Fig. 1H).

### IFNy treatment results in microglia with a more rounded morphology

**Figure 2:**
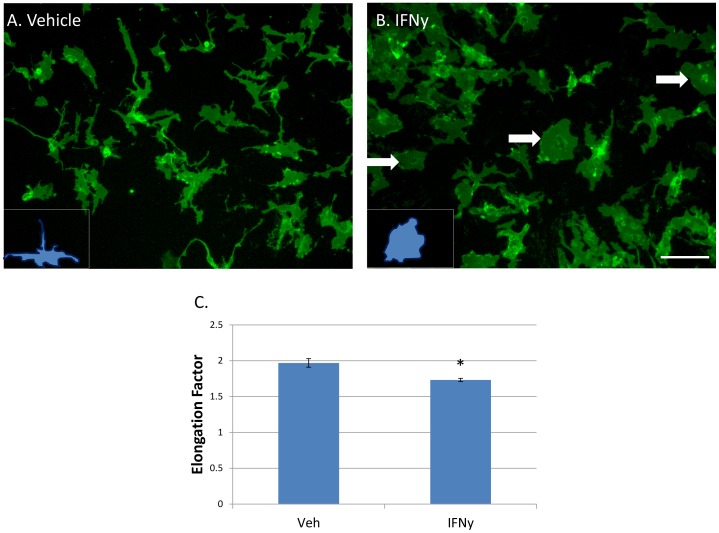
IFNy produces a change in microglia morphology to a more rounded, less elongated form. A) Adult human microglia immunolabelled with the cell surface marker CD45 have a heterogeneous morphology in basal conditions without any treatment. B) IFNy (1 ng/ml, 96 h) resulted in microglia with a rounder morphology (arrows). Insets in A) and B) show representative morphology of cells. Scale bar = 100 µm. C) Quantification of the ‘rounding’ effect using Metamorph *Elliptical Form Factor* (a measure of elongation) image analysis demonstrates a significant shift in microglia shape following IFNy treatment to a more rounded and less elongated form (N = 12).

The morphology of untreated adult human microglia *in vitro* is heterogeneous, with cells having variable protrusions and extensions. Microglial morphology is presumed to relate to their function, although exactly how is currently unclear. Round ‘amoeboid’ microglia are traditionally viewed as activated, inflammatory microglia [53]. We observed a quantifiable change in microglial morphology following 96 h IFNy treatment toward a rounded, less ramified shape (Fig. 2A and B). The ‘elongation’ of microglia was quantifiably reduced by IFNy as shown using the *Elliptical Form Factor* image analysis tool in MetaMorph software (Fig. 2C).

### Astroglial expression of HLA-DP, DQ, DR is increased by IFNy but not affected by TGFβ_1_ or M-CSF

**Figure 3:**
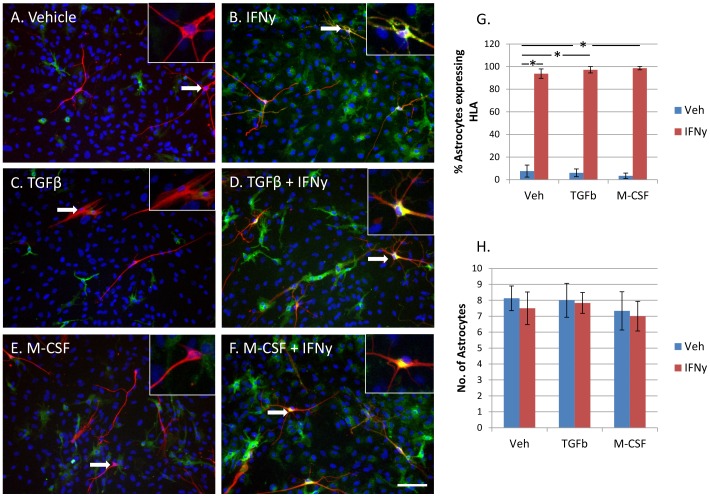
Astrocytic expression of HLA-DP, DQ, DR is increased by IFNy, and not changed by TGFβ_1_ or M-CSF. A) Adult human GFAP +ve astrocytes (red) express variable levels of HLA-DP, DQ, DR (green) in basal conditions without any treatment. B) IFNy (1 ng/ml, 96 h) increased astroglial expression of HLA-DP, DQ, DR. C) TGFβ_1_ (10 ng/ml) did not affect astrocyte HLA-DP, DQ, DR expression alone, or when enhanced by IFNy treatment (D). E) M-CSF (25 ng/ml) also did not affect basal HLA-DP, DQ, DR expression in astrocytes or IFNy-enhanced HLA-DP, DQ, DR expression in astrocytes (F). Insets show close-up examples of astrocytes indicated by arrows. Scale bar = 100 µm. G) A significant increase in percentage of HLA-DP, DQ, DR-immunopositive astrocytes is found with IFNy treatment. Neither TGFβ_1_ nor M-CSF significantly affect astrocyte HLA-DP, DQ, DR protein expression (N = 12). H) Quantification of GFAP-immunopositive astrocyte cell number (per well) following treatment with IFNy, TGFβ_1_ or M-CSF does not result in any significant differences compared to vehicle-treated cells (N = 12).

Astrocytes from different cases express differing basal amounts of HLA-DP, DQ, DR. From 10 biopsy cases, 2 had high basal astrocytic HLA expression, 3 had moderate expression and 5 had low or no HLA expression (Table 2). Basal astrocytic expression of HLA was generally higher when microglial HLA expression was high (Table 2), but the percentage of astrocytes expressing HLA (<10%) was lower than for microglia (40%, Fig. 1G and 3G).

Astrocytes were identified in human adult mixed glial cultures by expression of glial fibrillary acidic protein (GFAP). The number of astrocytes expressing HLA, and the amount of HLA expressed, was greatly increased in all cases by exposure to IFNy (Fig. 3A, B and G). TGFβ_1_ and M-CSF had no effect on IFNy-induced astrocytic HLA expression (Fig. 3D, F and G). TGFβ_1_ and M-CSF also did not influence basal HLA expression by astrocytes (Fig. 3C, E and G).

While IFNy increased HLA expression in astrocytes, it did not influence the number of GFAP-immunopositive astrocytes. TGFβ_1_ and M-CSF did not affect GFAP-immunopositive astrocyte cell number either (Fig. 3H).

### IFNy induces HLA-DP, DQ, DR expression in brain-derived pericytes

**Figure 4:**
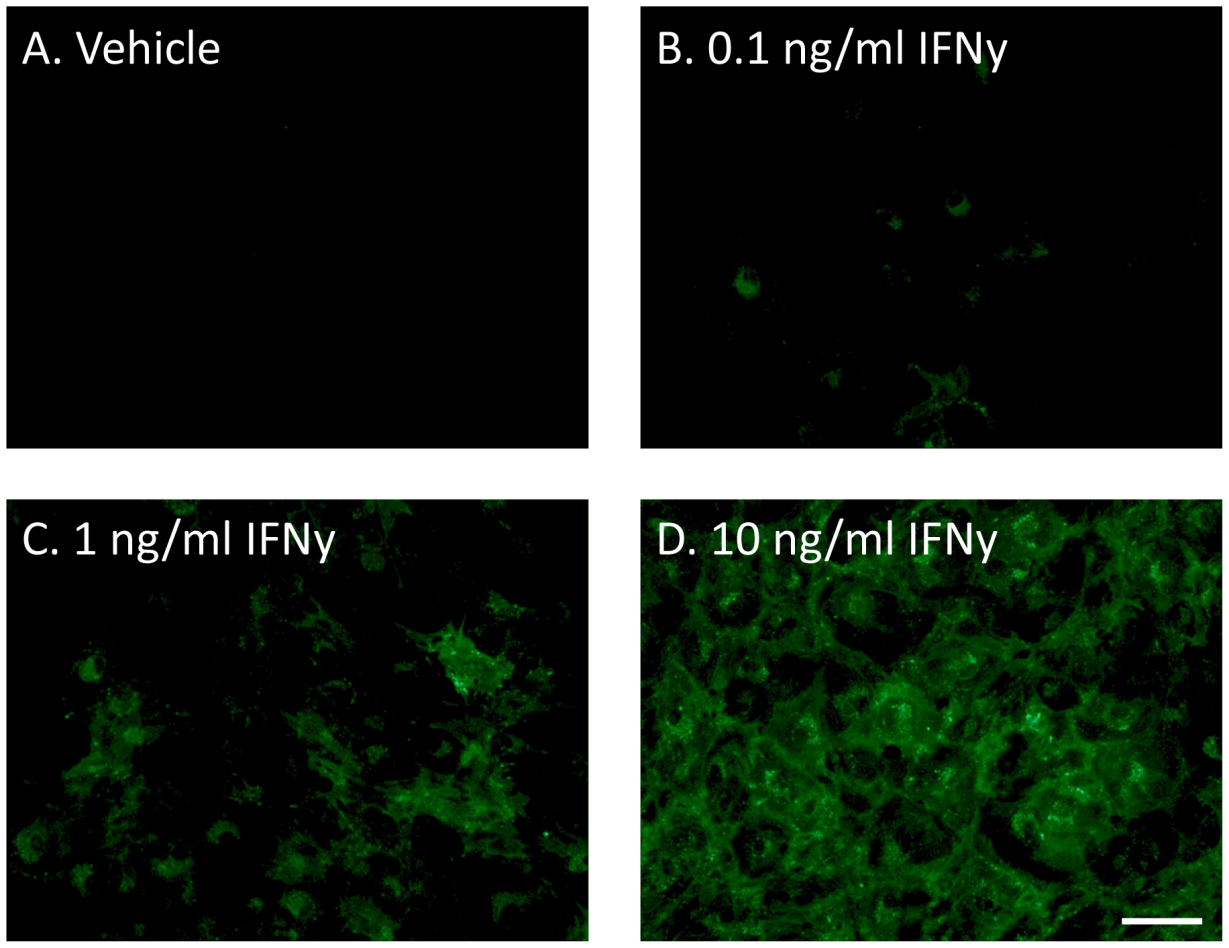
Brain-derived pericytes do not express HLA-DP, DQ, DR protein under basal conditions but it is induced by IFNy in a concentration-dependent manner. A) In normal culture conditions of DMEM/F12 +10% FBS +1% PSG brain pericytes do not express HLA-DP, DQ, DR protein. However, IFNy (0.1–10 ng/ml, 96 h) induced a concentration-dependent increase in HLA-DP, DQ, DR expression (B-D). Microglia and astrocytes are not present in cultures after 3–4 passages, producing a culture of pericytes only. Scale bar = 50 µm.

We next investigated HLA induction in the third population of cells in our mixed human glial cultures: pericyte cells [21], [22]. Pure cultures of brain pericytes were obtained after 3–4 passages of mixed glial cultures as they are the predominant cell type to divide basally in culture [21]. These cells do not express HLA basally without stimulation (Table 2 and Fig. 4A). However, upon exposure to IFNy they elicit a robust response by increasing HLA expression in a concentration-dependent fashion (Fig. 4). This response was seen for cultures of pericytes from both biopsy and post-mortem tissue from a range of neurologically diseased (Epilepsy, Alzheimer's, Huntington's and Parkinson's disease) and normal individuals. Pericytes had the same response whether in mixed cultures with microglia and astrocytes, or in cultures of pericytes alone.

### IFNy-induced pericyte HLA-DP, DQ, DR expression is inhibited by TGFβ_1_ but not by M-CSF

**Figure 5:**
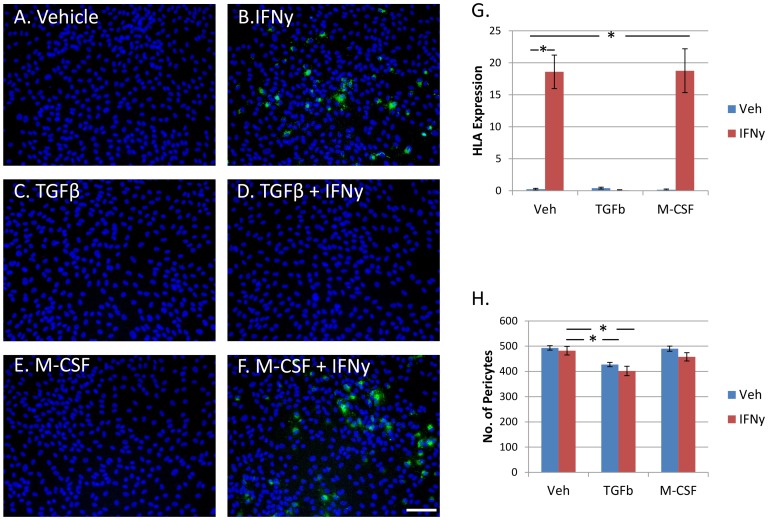
IFNy-induced expression of HLA-DP, DQ, DR in brain-derived pericytes is inhibited by TGFβ_1_ but not by M-CSF. A) Vehicle-treated pericytes (Hoechst-labelled nuclei) do not express HLA-DP, DQ, DR protein. B) IFNy induces a major up-regulation of HLA-DP, DQ, DR protein (green) in brain pericytes. C) TGFβ_1_ treatment alone does not induce expression of HLA-DP, DQ, DR in these cells. However, TGFβ_1_ completely inhibits the IFNy-stimulated increase in HLA-DP, DQ, DR (D). M-CSF affects neither basal (E) nor IFNy-induced (F) HLA-DP, DQ, DR expression in brain pericytes. Scale bar = 100 µm. G) HLA-DP, DQ, DR is induced by treatment with IFNy, and inhibited by simultaneous exposure to TGFβ_1_ but not M-CSF (N = 12). H) Pericyte cell number (per well) is not influenced by IFNy or M-CSF but is significantly decreased by TGFβ_1_ (N = 12).

Whereas no effect of TGFβ_1_ on HLA induction was seen for adult human microglia, there was a major inhibition effect of TGFβ_1_ on brain pericytes (Fig. 5D and G). This response was seen for pericytes alone and within mixed glial cultures with microglia and astrocytes present. Conversely, whereas microglial HLA induction was reduced by M-CSF, pericytes were unaffected (Fig. 5F and G). This is expected from previous findings of the M-CSF receptor (c-fms) being expressed only on microglia in primary human mixed glial cultures [49]. IFNy or M-CSF treatment had no effect on total number of pericytes as measured by Hoechst staining of nuclei in pericyte-only cultures (Fig. 5H). However, TGFβ_1_ was found to reduce pericyte cell number (Fig. 5H).

### IFNy also induces meningeal fibroblasts to express HLA-DP, DQ, DR

**Figure 6:**
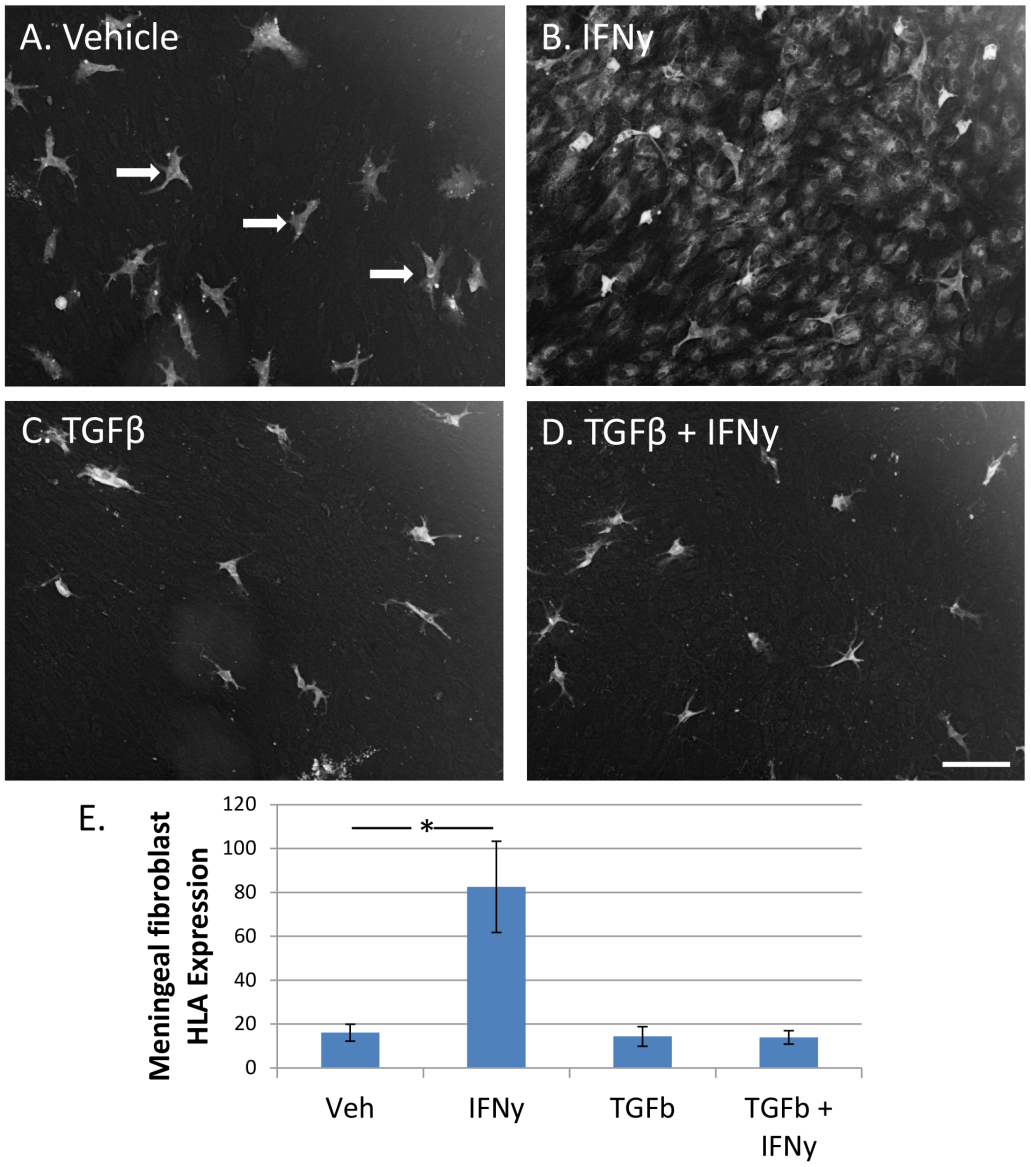
IFNy-induced expression of HLA-DP, DQ, DR in meningeal fibroblasts is completely blocked by TGFβ_1_. A) In vehicle-treated leptomeningeal explant cultures only leptomeningeal/perivascular macrophages express HLA-DP, DQ, DR (indicated by arrows). B) IFNy increases intensity of HLA expression on macrophage cells and greatly induces HLA expression in meningeal fibroblasts. C) TGFβ_1_ has no effect on basal leptomeningeal/perivascular macrophage or meningeal fibroblast HLA expression. D) However, TGFβ_1_ completely inhibits IFNy-induced meningeal fibroblast HLA expression, without affecting leptomeningeal/perivascular macrophage HLA expression. DAB brightfield images have been inverted for image analysis. Scale bar = 100 µm. E) Quantification of HLA expression shows a massive increase in HLA expression in leptomeningeal explant cultures with IFNy but not with TGFβ_1_ + IFNy (N = 12).

To study the induction of HLA-DP, DQ, DR in meningeal fibroblasts we undertook explant culture studies. Explant cultures were generated from leptomeninges overlying the middle temporal gyrus from both biopsy epilepsy specimens and autopsy specimens. The explant cultures generated cells over 1–2 weeks in 24-well plates. Once confluent, the explants were removed (and placed in a new 24-well plate) and the remaining adherent cells were characterised using antibodies to prolyl-4-hydroxylase and fibronectin for meningeal fibroblasts, and CD45 and PU.1 for leptomeningeal/perivascular macrophages. The majority of cells (>95%) were prolyl-4-hydroxylase and fibronectin-immunopositive meningeal fibroblast cells, with scattered CD45 and PU.1-immunopositive leptomeningeal/perivascular macrophages. HLA-DP, DQ, DR was absent in untreated meningeal fibroblasts, but present in leptomeningeal/perivascular macrophages (Fig. 6A). This pattern of staining matches closely that found in the pericyte cells and microglia derived from dissociated mixed glial cultures used in this study. IFNγ induced strong expression of HLA in meningeal fibroblasts (Fig. 6B and E). This expression was again completely blocked in meningeal fibroblasts by TGFβ_1_ (Fig. 6D). Interestingly, consistent with the failure of TGFβ_1_ to reduce microglial HLA expression, it also failed to reduce the expression of HLA in leptomeningeal/perivascular macrophages (Fig. 6).

### IFNy treatment of primary adult human mixed glia results in increased pro-inflammatory cytokine and chemokine release

**Figure 7:**
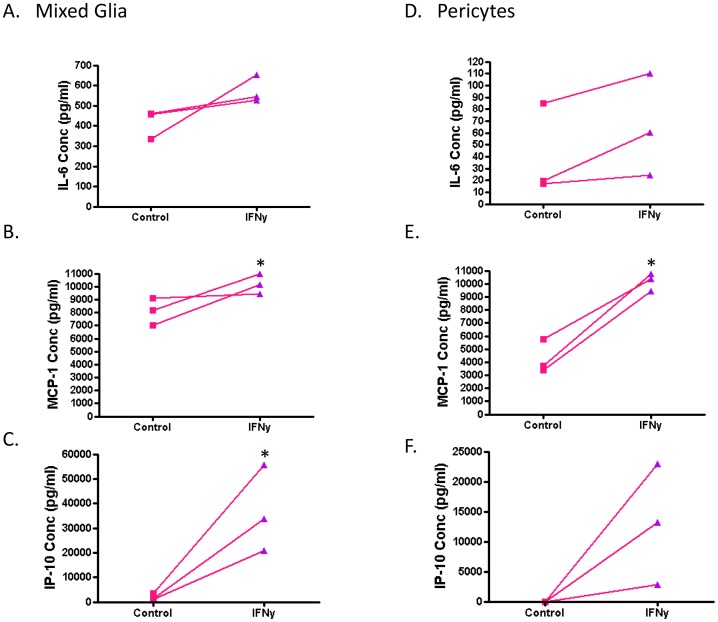
IFNy increases pro-inflammatory cytokine and chemokine release from adult human mixed glial cultures and brain-derived pericytes. Here each data point indicates an individual case (n = 3). Control and IFNy-treated samples from the same case are indicated by connecting lines. A) IL-6 secretion is slightly, but not significantly, increased by IFNy treatment of mixed glia (microglia, astrocytes and brain-derived pericytes). B) MCP-1 production is significantly increased by IFNy in mixed glia cultures. C) Adult human mixed glia produce a low basal level of IP-10 which is markedly increased by IFNy. D) Relatively low concentrations of IL-6 production by pericytes are not changed by IFNy. E) Pericyte cell cultures produce comparable levels of MCP-1 to mixed glial cultures when stimulated with IFNy. F) Pericytes release IP-10 upon IFNy stimulation only.

Another important function of microglia is production and secretion of cytokines. To assess the effect of IFNy on the production of these immune signalling molecules, we measured an array of cytokines and chemokines in the conditioned media of vehicle control and IFNy-treated mixed glial cultures (containing microglia, astrocytes and brain pericytes) using a Cytometric Bead Array (B.D Biosciences). A total of 16 cytokines was assessed, of which 10 (GM-CSF, IFNy, TNF, interleukin (IL)-1β, IL-2, IL-4, IL-5, IL-7, IL-12p70 and IL-13) were not detected in conditioned media from IFNy-treated, nor vehicle-treated, adult human mixed glia cultures. IL-10 and MIP-1α were detected at very low levels (<5 pg/ml) in both IFNy-treated and non-treated cells' conditioned media. IL-6, IL-8, IP-10 and MCP-1 were expressed at moderate levels in vehicle-treated cells' conditioned media (0.5–10 ng/ml). With IFNy treatment, there was no change in IL-8 concentration. IL-6 concentration was increased with IFNy (418±42 [mean ± SEM] pg/ml for vehicle treatment vs 576±39 pg/ml for IFNy treatment, n = 3; P = 0.0519), though not to statistical significance (Fig. 7A). MCP-1 concentration was significantly increased with IFNy (8104±608 pg/ml for vehicle treatment vs 10190±437 pg/ml for IFNy treatment, n = 3; P = 0.0493) (Fig. 7B). However, the biggest IFNy-induced change was an increase in IP-10 concentration from 2021±782 pg/ml for vehicle treatment to 36860±10140 pg/ml for IFNy treatment (n = 3; P = 0.0267) (Fig. 7C).

Cultures of pericytes alone (passage 5 – no microglia or astrocytes present as determined by immunolabelling) did not secrete IP-10 under vehicle conditions but did with IFNy (13003±5798 pg/ml, n = 3), albeit to a lesser extent than the mixed glial cultures (Fig. 7F). Pericyte-only cultures also had lower basal secretion of IL-6 and MCP-1 but whereas IL-6 concentration was not changed by IFNy treatment (Fig. 7D), MCP-1 concentration was increased as for mixed glial cultures (4293±735 pg/ml for vehicle treatment vs 10190±387 pg/ml for IFNy treatment, n = 3; P = 0.0021) (Fig. 7E).

**Figure 8:**
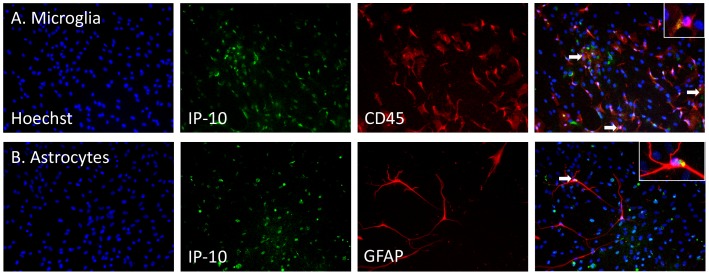
IP-10 is expressed by microglia and astrocytes in primary adult human mixed glial cultures. A) Following IFNy treatment (1 ng/ml, 96 h) IP-10 expression (green) is co-localised with CD45-immunopositive microglia (red). All nuclei are labelled with Hoechst (blue). Hoechst, IP-10 and CD45 and overlaid in the far right image. B) GFAP-immunopositive astrocytes (red) express IP-10 following IFNy treatment. Hoechst, IP-10 and GFAP are overlaid in the far right image. Scale bar = 100 µm. Arrows indicate high levels of IP-10 expression and insets show close-up examples of cells expressing IP-10.

**Figure 9:**
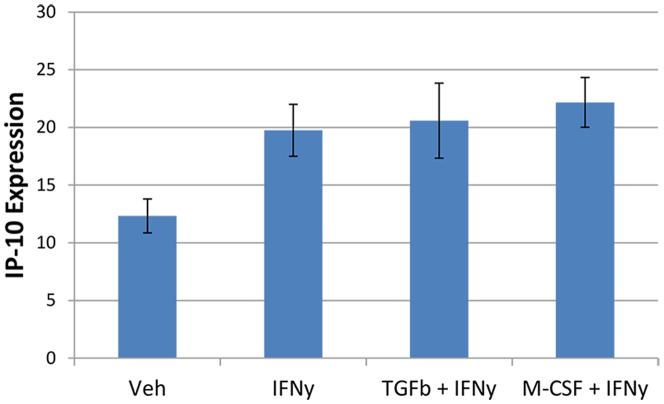
IFNy induces IP-10 expression in pericytes and is not affected by TGFβ_1_ or M-CSF. Following IFNy treatment, IP-10 expression is significantly increased in pericytes from basal levels. Simultaneous treatment with either TGFβ_1_ or M-CSF does not affect the levels of IP-10 expression induced by IFNy (N = 12).

The increase in IP-10 production with IFNy treatment can also be visualised by immunocytochemistry. This revealed that IP-10 is produced by both microglia and astrocytes in our mixed glial cultures and thus is likely to be secreted into the extracellular environment by multiple cell types (Fig. 8). In pericyte-only cultures, IP-10 staining is increased by IFNy. Unlike the inhibition of HLA expression by TGFβ_1_, the IFNy-induction of IP-10 expression was not blocked by TGFβ_1_ (Fig. 9). We did not observe an effect of M-CSF on IP-10 levels in pericytes either (Fig. 9).

## Discussion

Our findings demonstrate that HLA-DP, DQ, DR is an inducible protein which is not expressed constitutively by all adult human microglia, and that levels of HLA expression vary between individuals (Table 2). This study investigates glia from a number of neurologically diseased and normal human brains. However, no correlations were observed between particular disease states and neuroinflammatory protein expression. With the use of larger numbers of brains and a broader range of disease grades this information may be obtainable. Despite variable basal HLA expression, microglia from all cases consistently showed increased HLA with IFNy treatment (Fig. 1). In our studies we have used an antibody which targets HLA classes DP, DQ and DR. It will be interesting to see if the changes in expression we observed are due to one or more particular classes.

The results of the present study, together with previous work, suggest that the effects of TGFβ_1_ on IFNy-induced HLA expression are species specific as well as cell type specific. We found that TGFβ_1_ did not affect HLA expression in adult human microglia, either at basal levels or with IFNy-induction (Fig. 1). Conversely, TGFβ_1_ blocked IFN-y-induced enhancement of CIITA in murine macrophages and microglia [8], [30], [54], and human macrophage U937 cells [55]. This differential finding between rodent and human microglia is of major importance for understanding human neuroinflammation, especially given the emphasis in the literature of the anti-inflammatory properties of TGFβ_1_ [56].

**Figure 10:**
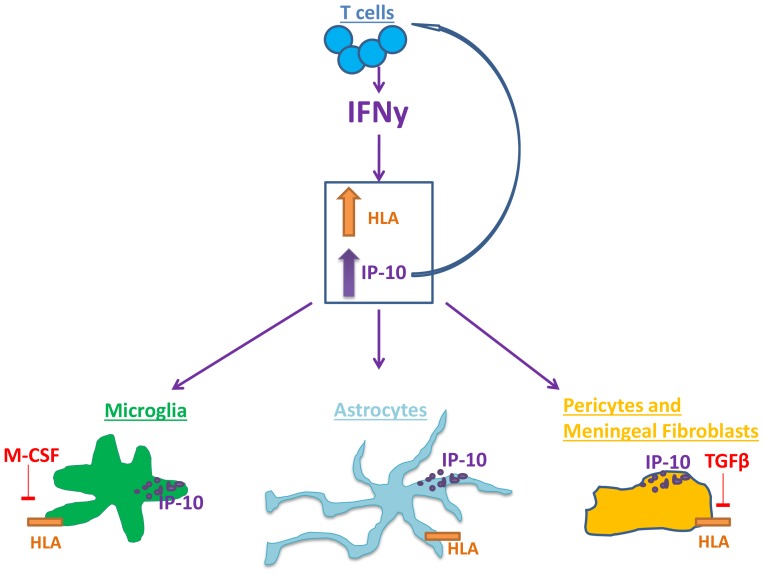
Differential regulation of HLA and IP-10 in adult human microglia, astrocytes, brain pericytes and meningeal fibroblasts by IFNy, TGFβ_1_ and M-CSF. The T cell pro-inflammatory cytokine IFNy upregulates HLA and IP-10 protein expression in adult human brain glial cells, pericytes and meningeal fibroblasts. Microglial HLA was increased by IFNγ (1 ng/ml for 96 h). M-CSF (25 ng/ml), but not TGFβ_1_ (10 ng/ml), was found to decrease microglial HLA expression. Astrocytic expression of HLA was also increased by IFNγ, and not modulated by TGFβ_1_ or M-CSF. Brain pericytes and meningeal fibroblasts do not basally express HLA but have a marked induction on exposure to IFNγ, which was blocked by TGFβ_1_. IFNγ increased adult human microglia, astrocyte and pericyte expression and release of pro-inflammatory cytokines and chemokines, particularly IP-10. IP-10 may be involved in leukocyte trafficking into the CNS.

We show that although TGFβ_1_ did not affect microglial HLA, M-CSF significantly reduced HLA expression by microglia (Fig. 1 and 10). The effect of M-CSF on basal and IFNy-induced HLA-DR has previously been investigated in human fetal astrocytes and microglia [34]. Similar to our results, they found reduced HLA-DR with M-CSF for microglia but not astrocytes [34]. It has been found that IFNy-mediated MHC-II induction in rodents was significantly muted in tumor microglia/macrophages compared with normal brain [57]. As M-CSF has been demonstrated to be upregulated in brain tumors [58], [59], it could be a possible mediator of decreased HLA expression within tumors.

Both M-CSF and IFNy increased the number of microglia in culture, although M-CSF had a greater effect (Fig. 1H). We and others have previously shown an increase in microglial cell number with M-CSF treatment [34], [49]. Given that M-CSF reduced microglial HLA expression whereas IFNy increases HLA, it was surprising to find a similar effect of M-CSF and IFNy on microglial cell number. M-CSF increases proliferation of adult human microglia [49] but this was not observed for IFNy (data not shown).

We found that TGFβ_1_ reduced microglia cell number and this could in fact be a mechanism by which TGFβ_1_ exerts anti-inflammatory effects. Previous reports in rodents have shown that TGFβ_1_ inhibits microglial proliferation [60], [61]. Our results show a similar effect of TGFβ_1_ on human and rodent microglial cell number, but a differential effect of TGFβ_1_ on microglial HLA expression.

Our immunocytochemistry and morphological analysis show increased rounding of IFNy-treated adult human microglia, with increased HLA-DP, DQ, DR staining (Fig. 2). Immunohistochemistry of brains of adult humans with MS has shown HLA-DR+ cells with oval morphology within MS lesions, whereas cells just outside the lesion and in the normal appearing parenchyma had a more ramified morphology [62]. Furthermore, expression of HLA class II molecules was noted to be less intensive on rod-shaped microglia compared to neighbouring ramified microglia in neurologically diseased human brain tissue [63]. To complement this finding we report here that IFNy treatment produces the opposite effect of rounded microglial morphology with increased HLA expression. ‘Activated microglia’ cannot be solely defined by morphology or expression of a single cell surface marker [11]. However, together with increased HLA-DP, DQ, DR and IP-10 expression, this change in morphology is suggestive of a pro-inflammatory microglial phenotype.

We also reiterate previous findings that HLA can be expressed by other brain cell types apart from microglia. We demonstrate that HLA is expressed by a small percentage of astrocytes under basal culture conditions and that they readily increase HLA expression upon IFNy stimulation (Fig. 3). Early studies of HLA-DR expression on cultured human adult astrocytes similarly found that a small proportion expressed HLA-DR and that there was a concentration-dependent increase in HLA-positive astrocytes with IFNy stimulation [62], [64]. While microglia are the predominant cell type to express HLA both *in vitro* and *in situ*, Styren et al. (1990) have shown that astrocytes in control and AD brains can express HLA-DR, although they are reported to be rare compared to HLA-DR-positive microglia [28].

Astrocytes were not responsive to either TGFβ_1_ or M-CSF when analysed for HLA expression (Fig. 3). We have previously reported that GFAP-positive astrocytes in mixed human adult glial cultures are negative for M-CSF receptor protein [49]. Astrocytes have however been shown to produce TGFβ_1_ and M-CSF which then act on other brain cells [34], [65]. These differential cell type responses to TGFβ_1_ and M-CSF show that astrocytes have a distinct immune phenotype and have an important role in brain immune responses.

A study investigating the expression of the IFNy receptor on human cells and tissue found astrocytes to be the predominant cell type with IFNy receptor expression [66]. Astrocytes, but not microglia or oligodendrocytes, expressed IFNy receptor in diseased and normal human brain tissue [66]. On the other hand, cultured human microglia, astrocytes and oligodendrocytes showed constitutive expression of IFNy receptor protein. While confirming IFNy receptor expression on microglia *in vitro*, this finding calls into question the physiological *in vivo* relevance of the effect of IFNy on microglia. However it will be important to confirm these immunohistochemical double-label results with *in situ* hybridization and a range of antisera to the IFNy receptor. If this result is validated by other studies it suggests that astrocytes are the main cells contributing to IFNy-mediated neuroinflammation in the brain.

The pericyte cell population did not express HLA in basal culture conditions, either in mixed glial cultures or in later passage (passage 3–5) pericyte-only cultures (Table 2). Exposure to IFNy resulted in a concentration-dependent increase in HLA expression by these brain pericyte cells (Fig. 4). These results show that brain pericyte cells have the capacity to be directed towards an immune role and may be an important target for treating neuroinflammation. Indeed, previous studies in rodents have shown that pericytes are involved in brain inflammation [18].

Despite TGFβ_1_ not affecting the microglial HLA response, TGFβ_1_ had a dramatic effect on HLA induction in brain pericytes (Fig. 10). TGFβ_1_ completely blocked IFNy-induced HLA expression in these cells (Fig. 5). Similar reports of TGFβ_1_ modulation of HLA expression have been made for human cells with fibroblast characteristics from other regions of the body [67], [68]. The finding that M-CSF does not influence brain pericyte expression of HLA is consistent with our previous observation that these cells don't express the receptor for M-CSF [49].

To further study the cellular basis of the induction of HLA in other non-glial cells, we undertook explant culture studies of leptomeninges tissue. These explants gave rise to cell cultures consisting predominantly of meningeal fibroblasts, with scattered leptomeningeal/perivascular macrophages. Leptomeningeal-explant derived meningeal fibroblasts responded to IFNy by expressing HLA in a similar fashion to dissociated brain pericyte cells (Fig. 6B). Furthermore, TGFβ_1_ abolished this induction (Fig. 6D and E). These results suggest that meningeal fibroblasts derived from leptomeninges and brain-derived pericytes respond in a common way to IFNy and TGFβ_1_, and indeed it is likely that our cultures from both dissociated brain tissue and leptomeninges contain mixtures of both cell types. As leptomeningeal/perivascular macrophages were also present in these cultures, it is possible that they can influence the response of meningeal fibroblasts to these cytokines, as might be expected *in vivo*.

The upregulation of HLA in individuals with neurological disease identifies HLA as an important molecule in the adult human brain, and one that may be important for communication with peripheral T cells. Increased intercellular adhesion molecule-1 (ICAM-1) expression has been found in epileptic and AD brains, and increased infiltration of CD8- and CD4- positive T lymphocytes was found in the hippocampus of patients with hippocampal sclerosis [69], [70]. ICAM-1 may aid T cell infiltration into the brain parenchyma where they could interact with antigen-presenting cells. However it is still unknown to what extent T cell activation occurs in the brain, and what factors govern this immune activation. The leptomeninges has been demonstrated to be a location of T cell contact with phagocytic antigen-presenting cells and a point of entry of encephalogenic T cells into the CNS [71], [72]. Live cell two-photon imaging of rats has revealed T cells moving out of leptomeningeal blood vessels and into the subarachnoid space where they interact with antigen-presenting cells and subsequently invade the CNS parenchyma [72]. In addition, the T cells became reactivated and upregulated pro-inflammatory cytokines and receptors including IFNy and CXCR3. Our data showing that both brain pericytes and meningeal fibroblasts can be induced to express HLA (as well as the chemokine IP-10) support this previous work and indicate that cells in the vasculature and meninges play major roles in brain inflammation.

Cytokines/chemokines are a major system of brain communication as there is mounting evidence that endogenous cytokines/chemokines in the brain act together with neurotransmitter and neuropeptide systems to control brain function [73]. We report extensive release of pro-inflammatory chemokines IP-10 and MCP-1 following IFNy treatment of adult human mixed glial cultures (Fig. 7B and C). IL-6 was present at lower levels under control conditions and not significantly increased by IFNy (Fig. 7A). Pure cultures of brain pericytes had lower basal cytokine/chemokine expression but also demonstrated a massive increase in MCP-1 and IP-10 release with IFNy treatment (Fig. 7E and F). Within mixed glial cultures the increase in MCP-1 may be largely from the pericytes as the relative increase in MCP-1 is much greater for pericyte-only cultures than for mixed glial cultures. Alternatively, the presence of microglia and astrocytes in mixed glial cultures may also be limiting MCP-1 release from pericytes. IL-6 levels were higher in the mixed glial cultures than in the pure pericyte cultures, suggesting that astrocytes or microglia are the main source of this cytokine. The increase in IP-10 release from mixed glial cultures is likely produced by all cell types present as we demonstrate immunocytochemical labelling of IP-10 production in microglia and astrocytes (Fig. 8), and pericyte-only cultures secrete IP-10 after IFNy treatment (Fig. 7F). Meningeal fibroblasts grown from explant cultures also expressed IP-10 in response to IFNy (data not shown), suggesting that meningeal fibroblasts are also a potential source of this chemokine in the inflamed brain.

IP-10 and MCP-1 can also be released by human fetal and simian adult astrocytes in response to IFNy [74]. Astrocytes and microglia have increased expression of IP-10 in several infectious and neurotoxic contexts including AD, ischemia and LPS-challenge [44], [75]–[77]. There is also evidence to suggest that not only glial cells but neuronal cells too can release chemokines to attract T cells into the brain parenchyma [78]. Adult human brain microvascular endothelial cells have been shown to upregulate IP-10 in response to IFNy [79]. The brain-derived pericytes and meningeal fibroblasts are also likely to be in ideal locations (i.e. blood vessels and leptomeninges) to convey systemic inflammatory signals to brain glia and neurons, acting as a gate-way between peripheral physiology and the CNS [80]. In cases of viral infection, Dionne et al. (2011) have demonstrated, using a brain slice culture model, that at least some of the IP-10 production and functional effects induced by viral infection are brain specific [81]. Interestingly, Durafourt et al. (2012) found IP-10 to be upregulated following activation in human microglia, but not in human macrophages, suggesting that IP-10 may be expressed by brain microglia more than peripheral macrophages in adult humans [82].

IP-10 expression is generally associated with loss of neuronal viability, however a direct mechanism has not always been established [78], [83]–[85]. As the IP-10 receptor CXCR3 is expressed by numerous cell types, IP-10 could act on a variety of cell types to eventuate in neuronal cell death. However, astrocytes and microglia have been found to respond differently to IP-10, and cellular background has been shown to determine CXCR3 signaling, highlighting cell type specificity in response to chemokines [39], [86].

IP-10 protein is expressed by macrophages in MS lesions and IP-10 and MCP-1 are expressed by astrocytes at the rim of MS lesions, while both microglia and astrocytes express the IP-10 and MCP-1 receptors CXCR3 and CCR2 respectively [43], [87]. CXCR3-positive astrocytes were also found to be increased in the CNS of HIV-positive patients, in ischaemic infarcts and in astrocytic neoplasms [13]. It has been suggested that IP-10-positive cells may represent a novel population of cells to target pharmacologically in a broad range of neurodegenerative conditions [88]. The effect of IP-10 on neuronal viability in the adult human brain remains unknown and pharmacologic reduction of IP-10 expression requires further exploration in the context of the adult human brain.

In conclusion, HLA and IP-10 are major players in neuroinflammation. Numerous studies have investigated their regulation, however few studies have been performed with human cells. This study used primary human adult glia to demonstrate species and cell type specificity in response to IFNy, TGFβ_1_ and M-CSF. While IFNy induced inflammatory responses in all human brain cell types studied, TGFβ_1_ and M-CSF have anti-inflammatory effects on specific cell populations (Fig. 10). In particular our studies have demonstrated that not only do the “classical” brain immune cells (microglia and astrocytes) show immune activation, but human brain pericytes and meningeal fibroblasts also show dramatic immune activation. This data is likely to have relevance for neuroinflammation in the adult human brain and more studies are warranted to determine the regulators of this neuroinflammation.
